# Gestational weight gain and postpartum weight retention in Tasmanian women: The Baby-bod Study

**DOI:** 10.1371/journal.pone.0264744

**Published:** 2022-03-22

**Authors:** Sisitha Jayasinghe, Manoja P. Herath, Jeffrey M. Beckett, Kiran D. K. Ahuja, Steven J. Street, Nuala M. Byrne, Andrew P. Hills

**Affiliations:** School of Health Sciences, College of Health and Medicine, University of Tasmania, Launceston, TAS, Australia; Public Library of Science, UNITED KINGDOM

## Abstract

Many factors can negatively impact perinatal outcomes, including inappropriate gestational weight gain (GWG). Despite having the greatest potential to influence maternal and infant health, there is a lack of consensus regarding the GWG consistent with a healthy pregnancy. To date, GWG in Northern Tasmania remains understudied. We investigated how maternal pre-pregnancy body mass index (BMI) is related to weight gain during pregnancy and weight retention post-partum, and how maternal pre-pregnancy BMI is related to the mode of delivery. Approximately 300 Tasmanian mothers (n = 291 for mode of delivery and n = 282 for GWG) were included in this study. Analysis of variance and chi square tests were conducted to assess differences in BW of mothers across BMI categories and differences between categorical variables; respectively. Based on pre-pregnancy BMI, mothers were assigned to one of three groups, with healthy weight (<25 kg m^-2^), with overweight (25–29.9 kg m^-2^), or with obesity (>30 kg m^-2^). Pre-pregnancy BMI and body weight (BW) were significantly associated (p<0.001) with post-partum BW at 3 and 6 months. Only 25% of mothers with a normal weight BMI, 34% with overweight and 13% with obesity, achieved the Institute of Medicine (IOM) recommendation for GWG. Interestingly, a number of women in our cohort lost weight during gestation (1.5, 9 and 37% in <25, 25–29.9 and >30 kg m^-2^ groups, respectively). Further, women with obesity showed the lowest level of BW fluctuation and retained less weight post-partum. The highest number of caesarean sections were observed in mothers who exceeded GWG recommendations. Most mothers either exceeded or failed to achieve IOM recommendations for GWG. To improve the generalisability of these findings, this study should be replicated in a larger representative sample of the Tasmanian maternal population.

## Introduction

Numerous factors can negatively affect perinatal outcomes, arguably unhealthy gestational weight gain (GWG) is one with the greatest potential to control [1]. Weight gain during pregnancy, in excess or deficit of the Institute of Medicine (IOM) recommendations, is associated with negative health consequences for both mother and newborn [2]. Despite generic recommendations, there is a lack of consensus in the literature regarding weight gain consistent with a healthy pregnancy. A recent report of a 16.5 year-observational cohort of ~60,000 singleton term live births reported that thinner women (body mass index [BMI] = 17 kg m^-2^) should be gaining more, and women with obesity (BMI = 32 kg m^-2^), should be gaining less than IOM recommendations [3]. Despite considerable evidence of links between maternal BMI and infant phenotypic outcomes, there is a paucity of information regarding optimal GWG in women at different BMI levels and early life impact on body composition [4].

Obesity is a major modifiable factor that compounds the GWG-perinatal outcomes dynamic. Australia ranks fifth among Organisation for Economic Co-operation and Development (OECD) countries for obesity with unhealthy levels of adiposity commonplace in females of childbearing age, along with inappropriate levels of GWG and post-partum weight retention [5]. Postpartum weight retention represents a significant preventable nutritional problem for women of reproductive age [6]. Literature is replete with evidence of weight retention between 1.5 and 3.0 kg, typically 12 months after parturition [7,8]. However, the amount retained has been reported to increase substantially (e.g., > 7.0 kg), in instances of higher than normal GWG [9]. In addition, numerous behavioural (e.g., sedentary time, physical activity, dietary and sleep patterns), psychological (e.g., maternal anxiety), and social factors (e.g., inadequate social support) have also been implicated in higher levels of postpartum weight retention [10].

Maternal obesity and excessive GWG are associated with increased rates of preeclampsia, gestational diabetes, birthing complications, premature birth and congenital abnormalities with consequent increased burden on personal and public health resources [11,12]. Tasmania has one of the highest rates of obesity in Australia [13]. Earlier infant health survey data highlighted an intergenerational relationship between maternal and infant body composition with elevated maternal obesity and higher infant skinfold thickness [14]. Further, Tasmania has higher perinatal deaths (10.9 vs 9.2 deaths per 1000 births) and pre-term births (10.2% vs 8.7%) compared with nationwide averages [13]. It is highly likely that endemic patterns of maternal obesity, GWG and perinatal outcomes in Tasmania are consistent with other Australian communities [15], however, GWG in Northern Tasmania remains understudied. The overall aims of this research were to (i) Investigate how maternal pre-pregnancy BMI is related to weight gain during pregnancy and weight retention post-partum, and (ii) investigate the relationship between maternal pre-pregnancy BMI and mode of delivery in a selected Tasmanian cohort.

## Materials and methods

The Baby-bod study is the Australian arm of a multi-centre international study to generate infant body composition reference data and extend the earlier work to generate infant and child growth standards based on the Multicentre Growth Reference Study [16]. As such, no *a priori* estimations of sample size were implemented. Participant recruitment and data collection in its entirety was conducted at the Launceston General Hospital, Tasmania, Australia, from September 2017 to October 2019. All procedures were approved by the Human Research Ethics Committee (Tasmania) Network; H0016117 and conformed to the guidelines of the National Health and Medical Research Council’s National Statement on Ethical Conduct in Human Research 2007 (Updated 2018). Participants provided written informed consent prior to being enrolled.

### Study population

Data from ~300 Tasmanian mothers (n = 291 for mode of delivery and n = 282 for GWG) were included in this report. Strict inclusion criteria implemented at recruitment included: 1. mothers who were ≥18 years of age and able to speak and understand English; 2. having a singleton pregnancy and 3. delivery at term. Women were excluded if they did not meet these criteria, if they presented with significant morbidities (as judged by attending clinician), or infants were born with a congenital anomaly. Some participant attrition was observed with approximately 60% and 55% of the originally recruited mother-baby dyads returning at 3 and 6 months, respectively.

### Outcome measures

Prenatal BMI was calculated using self-reported weight and height. Mothers were categorised according to World Health Organisation BMI classifications (underweight <18.5; healthy weight 18.5–24.9; overweight 25.0–29.9 and obese, >30.0 kg m^-2^). As only 2% (n = 8) of mothers were ‘underweight’ according to these guidelines, this group was combined with ‘healthy weight’ mothers for analytical purposes. Weekly GWG, total GWG and post-partum weight retention was enumerated as follows:

Post-partum weight retention (kg) 3 months = BW (at 3 months)–BW (pre-pregnancy)

Post-partum weight retention (kg) 6 months = BW (at 6 months)–BW (pre-pregnancy)

Weekly GWG (kg/week) = [net GWG ÷ self-reported gestational age]

Total GWG = [Weekly GWG *41(mean gestation in weeks)]

Excessive GWG gain was defined by the upper limit of IOM guidelines for each weight category (under/healthy weight >18 kg, with overweight >11.5 kg and with obesity, >9 kg).

### Body composition and anthropometric measurements

Maternal body weight and height measures (apart from prenatal weight which was self-reported), were completed in duplicate to maintain reliability at each of the visits. BW was recorded to the nearest gram using a digitized scale (SECA Corp. Hamburg, Germany) and height was measured to the nearest millimetre using a stadiometer (SECA Corp. Hamburg, Germany).

### Statistical analyses

All statistical analyses were conducted using the Statistical Package for the Social Sciences (SPSS) software (SPSS Version 26, Inc., Chicago, IL, USA). Results are presented as means and (95% CI), unless specified. A repeated measures analysis of variance was conducted to assess differences in BW of mothers across BMI categories. Statistical significance was set as *p*<0.05.

## Results

### Participant characteristics

According to self-reported (at interview in the maternity ward) ethnicity by the consenting mother, 1.5% of participants identified as Aboriginal or Torres Strait Islander, 8.5% as other (Hispanic, Asian, African, Middle eastern and Pacific Islander), and an overwhelming majority (~90%) as Caucasian. Mothers had an average age of 30 (range 18–48) years and a prenatal BMI of 27 kg m^-2^.

### Pregravid BMI, GWG and weight retention post-partum

**Fig 1:**
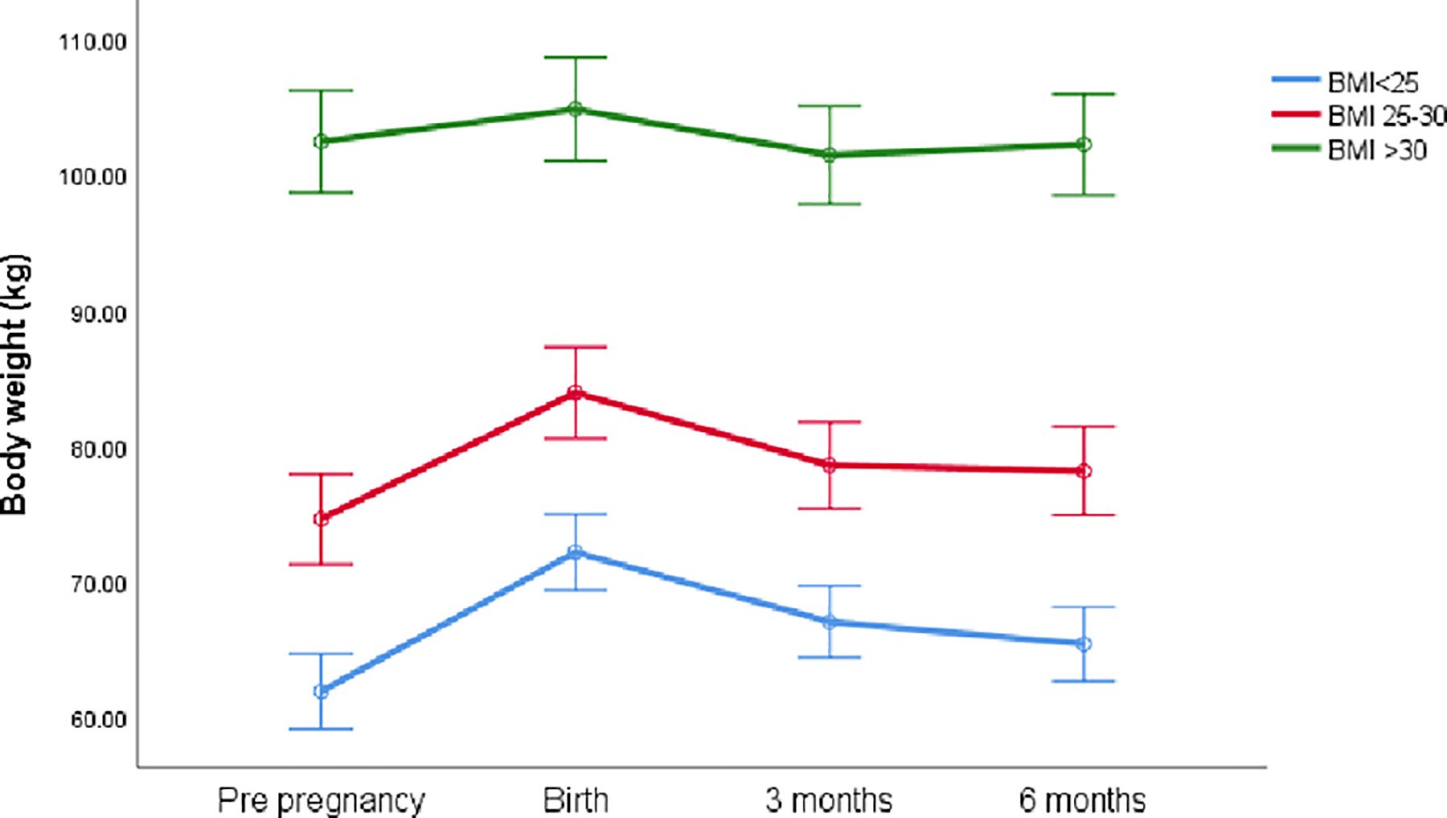
Pre- and post-partum BW of mothers from different BMI categories (mean ± SD).

**Fig 2:**
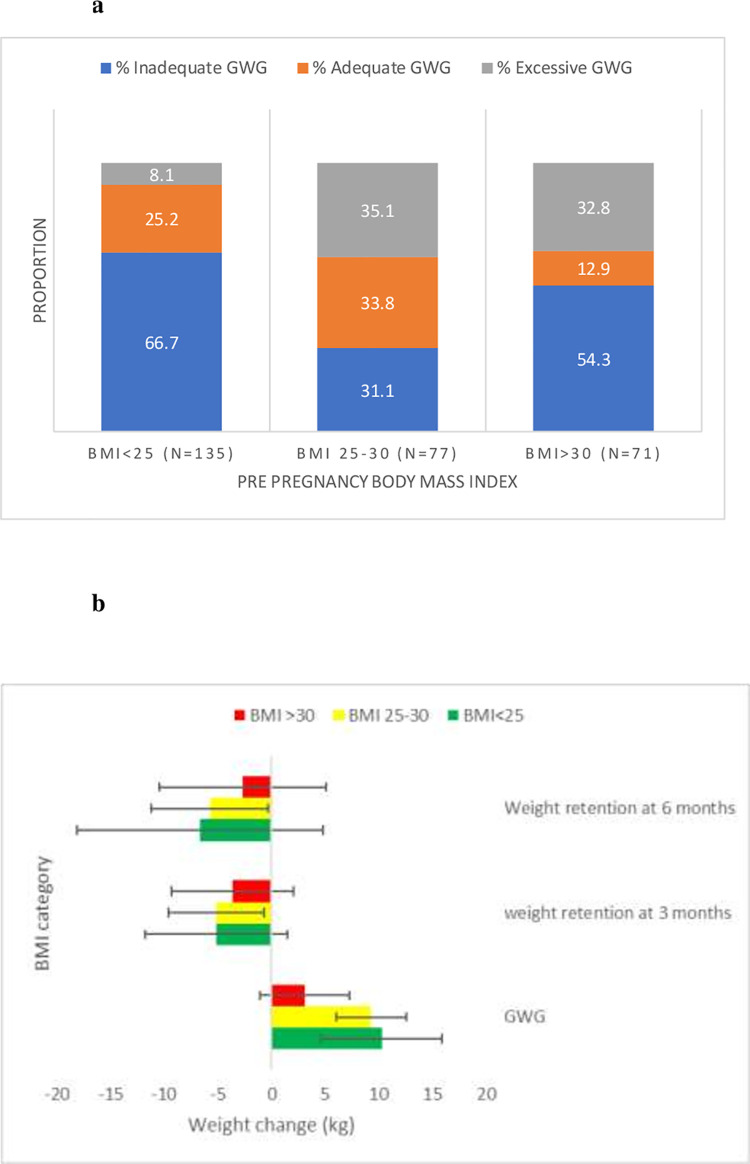
Adequacy of gestational weight gain (a) and post-partum weight retention (b) in Baby-bod mothers.

Repeated measures analysis of variance revealed significant changes in BW from conception to 6 months post-partum (Fig 1) in all mothers, regardless of BMI category (time, within and between-participant effects, *p*<0.001). GWG in approximately one-third of the mothers in the healthy weight and overweight BMI categories were consistent with IOM recommendations (Fig 2A). In the >30 kg m^-2^ BMI category, only 12.9% of women gained between 5–9 kg (Fig 2A). The lowest proportion of excess weight gain was observed in the <25 kg m^-2^ category (8.1%) with >30% of the mothers gaining in excess of the IOM recommendations in the other 2 groups (Fig 2A).

On average, women in the <25 kg m^-2^ BMI category gained the highest amount of weight during gestation (Fig 2B). Interestingly, women with obesity showed the lowest level of BW fluctuation and retained the least amount of weight post-partum (Fig 2B). When women were stratified based on absolute GWG, nearly 50% of women gained between 1–10 kg (Table 1). Of note, around 12% of women lost weight during their pregnancy and approximately 5% gained more than 20 kg (Table 1). In the category with obesity, approximately 40% of women lost weight during pregnancy whereas in the <25 kg m^-2^ category, only 2% lost weight during gestation. Moreover, 7% of women in this category gained more than 20 kg across the gestational period, the highest proportion among the three BMI categories (Table 1).

**Table 1:** Levels of gestational weight gain and birth outcomes of mothers based on different BMI (kg m^-2^) categories.

		BMI <25	BMI 25–30	BMI >30	Total
**GWG**	Lost weight	2 (1%)	7 (9%)	26 (37%)	35
	gained 1–10 kg	70 (52%)	34 (44%)	25 (36%)	129
	gained 10–20 kg	53 (13%)	34 (44%)	17 (24%)	104
	gained >20 kg	10 (7%)	2 (3)	2 (3%)	14
	Total	135	77	70	282
**Mode of delivery**	Vaginal spontaneous	84 (60%)	46 (57%)	40 (56%)	170
	Vaginal assisted	24 (18%)	10 (12%)	9 (13%)	43
	Caesarean section	31 (22%)	25 (31%)	22 (31%)	78
	Total	139	81	71	291

BMI, body mass index; GWG, gestational weight gain; *percent value indicates the proportion of women/births within the BMI category.

### Pregravid BMI and mode of delivery

Vaginal spontaneous delivery was more common in women with a normal weight BMI (60%) and least common in women with obesity (Table 1). A total of 78 caesarean sections were recorded across the cohort and collectively, women with overweight and obesity accounted for 60% (25 and 22, respectively) of caesarean sections (Table 1). The impact of GWG was pronounced in relation to spontaneous vs. assisted deliveries, with approximately 60% of the former in women who gained between 1–10 kg (Table 2). In women whose GWG was 10–20 kg, this proportion remained for mothers with a normal BMI but decreased substantially for women with a higher BMI (Table 3). For example, in the <25 kg m^-2^ category, mothers who gained in excess of 20 kg, 70% of deliveries were assisted vaginal or caesarean sections (Table 2). The proportion of caesarean sections was highest (35%) in women who gained more than IOM recommended weight ranges, while in comparison, only 25% of women who gained less than, or met the recommendations, had caesarean deliveries (Table 3).

**Table 2:** Birth outcomes stratified according to pre-pregnancy BMI and absolute GWG.

		Vaginal spontaneous	Vaginal assisted	Caesarean section	Total
Lost weight	BMI <25	2 (1%)	0	0	2
	BMI 25–30	5 (3%)	0	2 (2%)	7
	BMI >30	16 (10%)	2 (5%)	8 (11%)	26
Gained 1–10 kg	BMI <25	43 (26%)	10 (24%)	17 (23%)	70
	BMI 25–30	21 (13%)	5 (12%)	8 (11%)	34
	BMI >30	16 (10%)	2 (4%)	7 (9%)	25
Gained 10–20 kg	BMI <25	35 (21%)	8 (19%)	10 (14%)	53
	BMI 25–30	16 (10%)	4 (9%)	14 (19%)	34
	BMI >30	6 (4%)	5 (12%)	6 (8%)	17
Gained >20 kg	BMI <25	3 (2%)	5 (12%)	2 (2%)	10
	BMI 25–30	0	1 (1%)	1 (1%)	2
	BMI >30	2 (1%)	0	0	2
	Total	165	42	75	282

BMI, body mass index; GWG, gestational weight gain.

**Table 3:** Birth outcomes stratified according to adequacy of GWG and BMI categories.

		Vaginal Spontaneous	Vaginal Assisted	Caesarean section	Total
Inadequate weight gain	BMI <25	59 (36%)	11 (26%)	20 (27%)	90
	BMI 25–30	17 (10%)	2 (5%)	5 (7%)	24
	BMI >30	24 (15%)	3 (7%)	11 (15%)	38
Adequate weight gain	BMI <25	21 (13%)	6 (14%)	7 (9%)	34
	BMI 25–30	12 (7%)	5 (12%)	9 (12%)	26
	BMI >30	6 (4%)	1 (2%)	2 (3%)	9
Excessive weight gain	BMI <25	3 (2%)	6 (14%)	2 (3%)	11
	BMI 25–30	13 (8%)	3 (7%)	11 (25%)	27
	BMI >30	10 (6%)	5 (12%)	8 (11%)	23
	Total	165	42	75	282

BMI, body mass index; GWG, gestational weight gain.

## Discussion

This research investigated the impact of maternal pregravid BMI on longitudinal changes in BW (i.e., GWG and weight retention) and mode of delivery in a selected Tasmanian cohort. Only 25% of mothers with a normal weight BMI, 34% with overweight and 13% with obesity, achieved the IOM recommended levels of GWG. As such, an overwhelming majority of mothers either exceeded, or did not achieve, the currently recommended GWG levels, a pattern consistent with a recent report based on a marginalised Australian aboriginal cohort [17]. It is difficult to distinguish the specific causes of GWG outcomes in our cohort; however, ignorance of the IOM recommendations could be a potential contributor. Previous evidence indicates significant inaccuracies or misconceptions about adhering to GWG goals amongst Australian pregnant women. These trends may be further exacerbated by the low levels of health literacy reported in Australia [18]. Nonetheless, our data show that Tasmanian mothers with overweight or obesity had a higher propensity to gain excessive weight (~33% vs 8%) compared with mothers with a BMI of <25 kg m^-2^, a pattern of GWG consistent with recent Australian data [19].

There was a relatively high prevalence of inadequate GWG in our study with women with overweight being the only group, on average, to achieve the recommended GWG (mean GWG = 9.27 kg). To date, there is no global consensus regarding appropriate GWG, particularly for women with obesity [20]. Nevertheless, the literature is replete with higher than recommended gains and links with complications including preeclampsia, caesarean section, and macrosomia, with less than recommended GWG generally not linked with adverse outcomes [21]. Given the lack of consensus regarding GWG, it could be argued that clinical judgement should occur on a case-by-case basis with intervention (or not) implemented based on the quality of the growing foetus rather than fluctuations in weight. Some empirical evidence suggests a link between inadequate GWG and heightened incidence of small-for-gestational age (SGA) babies [22]. However, in the current study, only a minority (~5%; mean BW 2.4 kg) of babies were ‘small’ despite the majority of mothers failing to gain the recommended amount of weight.

Furthermore, some of the women in our cohort lost weight (1.5, 9 and 37% in <25, 25–29.9 and >30 kg m^-2^ groups, respectively) during gestation. Although the IOM does not advocate gestational weight loss, it is not uncommon for women to lose weight during pregnancy, and some women intentionally attempt to reduce body weight during this crucial phase of life [23]. Evidence suggests that for women with obesity, losing some weight during gestation is beneficial in reducing the risk of preeclampsia, caesarean sections and large-for-gestational age (LGA) babies [24]. Controversially, it has been suggested that a reduction of ~5 kg during gestation may be beneficial in more severe levels of obesity (i.e., Classes II and III) [25]. However, the benefits of gestational weight loss to maternal health and maternal birth outcomes need to be balanced against the increased risk of SGA neonates (2).

Metabolic and physiological changes during gestation impact maternal health and throughout later life [26]. For example, GWG in excess of the IOM guidelines is associated with a 3–5 kg retention of weight in the first 15 years after childbirth [27]. In the current study, we observed significant positive associations between both maternal pregravid weight status, GWG and post-partum weight retention. Currently, there is also no consensus on whether pre-pregnancy weight status or GWG is more influential in determining the level of post-partum weight retention [28]. Nevertheless, if retained for an extended period, weight gained during pregnancy can contribute to obesity and other metabolic anomalies in later life [29]. Although inconclusive, several physiological adaptations related to offspring sustenance (e.g., increased absorption of nutrients, hyperphagia, reduction in faecal excretion) are thought to be contributing to the overall weight retention after childbirth [30]. Pre-clinical evidence indicate that substantial changes in intestinal anatomy (i.e., weight and surface area) during pregnancy and lactation, and subsequent increases in nutrient uptake are significant contributors towards weight retention [31]. Although the optimal strategy for reducing post-partum weight is yet to be identified, existing literature suggests that a combined approach of diet and physical activity-related lifestyle changes is the most effective [28].

Our findings concur with evidence that excessive GWG is associated with higher rates of caesarean section [32]. Pre-pregnancy BMI is also reported to be associated with increased risk of caesarean section [33], and independent of mode of delivery, women with obesity are also twice as susceptible to post-partum haemorrhage than mothers with a normal BMI [34]. Although pertinent information is unavailable in the current instance, extant literature a variety of ‘indications’ for caesarean sections including dystocia, foetal distress, breech presentation, placental abruption/ praevia and cord prolapse [35].

The causal link and mechanistic associations between higher maternal BMI, obesogenic intra-uterine environment and subsequent infant overweight/obesity, is a fiercely debated topic [36]. A substantial body of research has exemplified how factors such as maternal pre-pregnancy weight, BMI and GWG can determine the quality of the intra-uterine environment and influence neonatal phenotype at birth [1,14]. Generally, mothers with obesity are 2–3 times more likely to deliver a LGA or macrosomic baby [37]. It is also important to highlight the significant negative impact of increased BMI and GWG on maternal wellbeing during pregnancy. Mothers with obesity have a 5–6 times higher likelihood of developing conditions such as preeclampsia and GDM compared with mothers with a normal body weight. Development of these conditions during gestation could be considered a ‘double whammy’ as a dual diagnosis of preeclampsia and GDM could increase a woman’s chance of developing diabetes in later life by about 18 times [38]. Taken together, the extant evidence suggests interventions focused on improving diet and exercise in women of childbearing age with a view to stabilising BMI within the healthy range, should be undertaken. A specific focus on healthy weight reduction in obese women before they become pregnant should be a public health priority.

## Conclusions

Overall, an overwhelming majority of mothers in this study either exceeded, or did not achieve, the currently recommended GWG levels. This, and similar trends reported in Aboriginal Australians [17], highlight the urgency of the public health response needed to optimize GWG. It was also noticeable that accretion of excess gestational weight is linked with the requirement of medical intervention at delivery. The ability to extrapolate findings to the wider Tasmanian population is somewhat limited as ethnicities other than Caucasian were underrepresented in our cohort. Given the unique patterns of GWG reported in various ethnic groups [39], it may be worthwhile to repeat this research in a more representative sample. It is also important to highlight that although self-report is a widely utilized, cost-effective and practical measurement approach, it can be prone to bias. Whilst the findings of this study add to the national and international evidence base regarding weight trajectory and health status of women during pregnancy, future research should try to circumvent the imprecise nature of a dependence on maternal recall to calculate GWG. Potentially, cohorts with a known date of conception (e.g., women in in-vitro fertilization clinics), along with novel approaches to monitoring body composition change with body weight change, may provide greater clarity. Efforts should also be made to assess factors other than GWG including dietary and physical activity habits, which may influence the weight retention after parturition.

## Supporting information

S1 File Baby-bod cohort data. (SAV)
